# Photosensitizing systems based on alginate aerogels and methylene blue for controlled release of dye for antimicrobial photodynamic therapy

**DOI:** 10.3389/fchem.2025.1702876

**Published:** 2025-11-11

**Authors:** Ilya Shershnev, Anna Solovieva, Alexander Kopylov, Anastasiya Cherkasova, Vladislav Kaplin, Sergey Rachev, Anastasiya Kuryanova, Nadezhda Aksenova, Victoriya Timofeeva, Anastasiya Akovantseva, Tatyana Zarkhina, Viktor Shantarovich, Valentin Bekeshev, Polina Bikmulina, Ru-Lin Huang, Qingfeng Li, Peter Timashev

**Affiliations:** 1 N.N. Semenov Federal Research Center for Chemical Physics, Russian Academy of Science, Moscow, Russia; 2 Institute of Fine Chemical Technologies, Russian Technological University, Moscow, Russia; 3 Institute for Regenerative Medicine, Sechenov University, Moscow, Russia; 4 Department of Plastic and Reconstructive Surgery, Shanghai Jiao Tong University School of Medicine, Shanghai 9th People’s Hospital, Shanghai, China

**Keywords:** calcium alginate, alginic acid, aerogels, methylene blue, photodynamic therapy, singlet oxygen

## Abstract

Photosensitizing systems based on methylene blue (MB)-loaded calcium alginate (CaA) and alginic acid (AA) aerogels were developed for photodynamic therapy of difficult-to-heal wounds. Hybrid aerogels incorporating polyvinylpyrrolidone (PVP, 2.5–40 wt%) into CaA and AA matrices were also made. The MB release kinetics in a phosphate buffer were found to depend on the aerogel type (AA or CaA). The incorporation of PVP increased the MB release rate by 1.5–2 times. The singlet oxygen (^1^O_2_) generation efficiency of MB embedded in the aerogels was influenced by their porosity and chemical composition. The activity of MB in the photogeneration of ^1^O_2_ increased by up to four times in the PVP-containing aerogels. Furthermore, the photoactivity of MB in the hybrid aerogel matrices significantly exceeded that in the single-component alginate aerogels.

## 1 Introduction

Aerogels based on polysaccharides (sodium alginate, chitosan, cellulose, etc.) are currently used in the medical, food, cosmetic, and pharmaceutical industries (Muhammad et al., 2021; Smirnova, 2011). Due to their high biocompatibility, biodegradability, and low antigenicity, aerogels are used in medicine as carriers of bioactive substances and drugs, in tissue engineering as matrices for the growth and differentiation of various cell types, and in the production of wound-healing coatings (Guastaferro et al., 2021; López-Iglesias et al., 2019; Souto-Lopes et al., 2024).

Currently, the problem of treating purulent wounds remains very relevant, despite the variety of treatment methods used in purulent surgery (Tedesco et al., 2025). Further improvement of methods for treating local purulent diseases of soft tissues and their complications remains one of the relevant areas of modern practical surgery. It is known that traditional methods of preventing and treating wound infections using antibiotics have not justified themselves. This is explained by the high rate of evolution of wound microflora with changes in their biological properties and the rapid development of resistance to antibacterial drugs (Murray et al., 2022). A full-fledged alternative to antibiotic therapy in wound treatment can be photodynamic therapy (PDT), a method related to laser technologies in medicine and developed in the 1960s for the diagnosis and treatment of tumors (Correia et al., 2021). To implement PDT, a photosensitizer (PS) (a substance that increases the sensitivity of tissues to light and selectively accumulates in cancer cells), and light with a wavelength that coincides with the absorption band of the PS are needed. It was later discovered that, in addition to cancer cells, many biological objects (bacteria, viruses, and fungi) can accumulate PS, as a result of which microorganisms become sensitive to low-intensity laser radiation of the corresponding wavelength (Rajabov et al., 2017). This, in turn, initiated developments in the photodynamic inactivation of bacteria, or antimicrobial photodynamic therapy (aPDT) (Almenara-Blasco et al., 2024; Hu et al., 2018).

Polysaccharides, especially sodium alginate (SA) and chitosan, are traditionally used as components of wound-healing agents or individually, in the form of wound dressings (Peng et al., 2022). Typically, polysaccharide coatings that form gels on the wound surface and contain antimicrobial agents are supposed to regulate wound moisture. However, as it was shown previously, such gel coatings do not have gas permeability, which leads to deterioration of wound ventilation (Han et al., 2023). A special type of solid polysaccharide gel, aerogels, has a highly developed surface with a unique system of nanosized (meso)pores (Lovskaya et al., 2015), which can provide a solution to the above problems. Aerogels are nanostructured mesoporous materials characterized by low density (0.003–0.35 g/cm^3^), large internal surface area (up to 1,000 m^2^/g), and pore sizes of several nanometers. The most studied aerogels, which are used in many industries, are based on silicon dioxide, metal oxides, and graphene (Kim et al., 2023; Menshutina et al., 2022; Niculescu et al., 2024). At the same time, aerogels on the basis of biologically active polysaccharides allow the formation of biocompatible mesoporous systems for use in pharmaceuticals as a carrier of biologically active substances (Solovieva et al., 2017).

In this work, aerogels based on sodium alginate (SA) were used as a matrix for the immobilization of methylene blue (MB), a dye with photosensitizer properties applied in aPDT. Because it was previously shown that the presence of polyvinylpyrrolidone (PVP) increases the activity of MB in the photogeneration of singlet oxygen, the dye was also introduced into hybrid aerogels based on SA and PVP. The effect of PVP on the porosity of the aerogels, the kinetics of dye diffusion into a phosphate buffer solution, and the activity of immobilized MB molecules in the photogeneration of singlet oxygen were studied. Such systems allow for controlled release of MB into the wound cavity and uniform distribution of the dye throughout the wound area, which increases the effectiveness of photodynamic action. At the same time, the alginate matrix can additionally have a bactericidal effect on the wound surface.

## 2 Materials and methods

### 2.1 Chemicals and materials

The following chemicals and materials are used in this manuscript:

CaA - Calcium alginate
AA - Alginic acid
MB - Methylene blue
PVP - Polyvinylpyrrolidone
sc-CO_2_ - Supercritical carbon dioxide
SA - Sodium alginate
PBS - Phosphate-buffered saline
MSC - Mesenchymal stromal cells
SDS - Sodium dodecyl sulfate

Aerogels were prepared using sodium alginate with a molecular mass (MM) of 150–300 kDa (Ruschem). Sodium alginate was treated with hydrochloric acid (Ruschem) or calcium chloride (granulated, Ruschem). The treatment methods are given below. Methylene blue (MB, 3,7-bis(dimethylamino)phenothiazin chloride, analytical grade, Chimmed) was introduced into the aerogels. Aerogels based on alginic acid (AA), calcium alginate (CaA), and mixtures of polysaccharides with polyvinylpyrrolidone (PVP, Sigma-Aldrich, MM 40 kDa) were used to immobilize the MB.

### 2.2 Methods for obtaining alginate aerogels

#### 2.2.1 Sodium alginate-based aerogels containing MB

A 2 wt% aqueous solution of SA was treated with hydrochloric acid (1 M) or a 5 wt% solution of calcium chloride, kept for 24 h until a hydrogel was formed (in the form of films), and then washed with water. Wet films of CaA or AA hydrogels were placed in an aqueous solution of MB and kept for a day, after which they were washed with an aqueous solution of isopropyl alcohol, with a gradual increase in the content of С_3_Н_8_О until the water in the pores of the gel was completely replaced by alcohol (Solovieva et al., 2017). To prevent dye removal from the hydrogel, the isopropanol contained MB. The resulting AA/MB and CaA/MB alcogels were dried using sc-CO_2_ for 6 h (130 bar, 50 °C), resulting in AA/MB and CaA/MB aerogels.

#### 2.2.2 Hybrid aerogels containing MB based on SA + PVP

A joint aqueous solution of sodium alginate (2 wt%) and PVP (5 × 10^−4^ M) was treated with hydrochloric acid (1 M) or a 5 wt% solution of calcium chloride, kept for 24 h at room temperature until a hydrogel was formed, and then washed with water. Films of AA/PVP or CaA/PVP hydrogels were placed in an aqueous solution of MB and kept for 24 h. The resulting AA/MB/PVP and CaA/MB/PVP hydrogels were processed according to Section 2.2.1, resulting in AA/PVP/MB and CaA/PVP/MB aerogels. All aerogels were films with a thickness of 100–1,000 μm.

The content of PVP (by nitrogen content) and residual sodium in aerogels was determined using elemental analysis. The nitrogen content was determined on a PerkinElmer 2400 CHN analyzer (United States), with a determination error of 0.3%; the sodium content was evaluated on a BWB-XP flame photometer (Great Britain). The MB content in aerogels was determined spectrophotometrically after complete release of MB from the sample in buffer.

### 2.3 Determination of porosity and swelling of aerogels

The specific surface area and parameters of the porous structure of the samples (with characteristic pore diameters in the range from 2 nm to 100 nm) were determined by low-temperature nitrogen sorption (77.4 K) on a NOVA 1200e gas sorption analyzer from Quantachrome Instruments (United States). Highly purified nitrogen gas was used as the adsorbate. Before the measurements, the samples were degassed in a vacuum for 3 h at 50 °C. The specific surface area was determined using the Brunauer–Emmett–Teller (BET) method in the relative pressure range *P*/*P*_0_ = 0.05–0.21 (P_0_ is the saturated vapor pressure of the adsorbate at the experimental temperature).

It should be noted that according to the classification of the International Union of Pure and Applied Chemistry (IUPAC), all pores are divided into three classes by size: macropores are pores with sizes exceeding 50 nm (some sources indicate a limit of 150–200 nm), mesopores are pores with sizes in the range from 2 nm to 50 nm, and micropores are pores with sizes less than 2 nm.

A positron annihilation method based on measuring the lifetime of positronium atoms was used to acquire information about pores with sizes smaller than 2 nm, characterizing the “free volume of the polymer,” which facilitates the processes of vapor and gas permeability and ensures ventilation of wounds. Positronium is formed in a condensed medium under positron irradiation, diffuses in the medium until localization in a region of reduced electron density, that is, in a pore, and perishes during annihilation. Measuring the lifetime *τ*_*i*_ of a positronium atom relative to annihilation in a pore allows one to estimate the effective radius of a micropore *R*_*i*_. The positron source was the radioactive isotope ^44^Ti. Measurements were performed using an EG&G ORTEC (United States) annihilation spectrometer with a time resolution (full width at half maximum (FWHM) of the prompt coincidence peak) of 0.3 ns.

The swelling of alginate aerogels in the absence and presence of PVP in a buffer solution (PBS) was studied. The mass of fully dried aerogel samples was measured and immersed in PBS at room temperature (25 ± 0.5 °C). After specified time intervals (5–60 min), the aerogel samples were removed from the buffer, and excess liquid was carefully removed with filter paper. The aerogels were then weighed on a laboratory scale Sartorius CP124 S (Germany) with an accuracy of ±0.1 mg.

The swelling ratio (*Q*) was calculated using the following equation:

$$Q=\frac{m_{t}-m_{0}}{m_{0}}\times 100\%$$

where *m*_0_ is the mass of the dry sample; *m*_t_ is the sample mass at the specific time point.

### 2.4 Diffusion of the dye from the matrices

The process of diffusion release of MB from aerogel matrices based on AA and CaA was studied using phosphate buffer solution (PBS, pH = 7.2) from Eco-Service (Russia) as a medium. The kinetics of MB release into PBS were studied by placing an aerogel film (∼0.02 g) containing MB in a thermostatted cell (37 °C), adding 10 mL of buffer solution, and stirring on a magnetic stirrer at 200 rpm. Complete release of the dye from the matrix into the buffer solution was achieved in 30–210 min, depending on the aerogel structure.

The results of measuring the optical density *D* of the solution (at *λ* MB = 665 nm), characterizing the content of methylene blue released from the polymer matrix into the PBS solution during diffusion over time t, are presented as a concentration dependence *C(t)*. The absorption spectra were recorded using a Cary 50 spectrophotometer from Varian (United States). The measurement error did not exceed 5%. The amount of the introduced dye was determined spectrophotometrically by measuring its concentration after the dye had completely released into the solution (from 30 min to 210 min). All dependencies demonstrated the first-order kinetics at the initial stages of the process; at time intervals corresponding to the release of 30%–50% of the amount of MB initially contained in the sample into the solution:

$$C\left(t\right)=C_{\text{tot}}\times \left[1-\exp\left(-k\times t\right)\right]$$

so that when *C*(*t*)<<*C*_tot_.

$$\frac{C\left(t\right)}{C_{\text{tot}}}∼k\times t$$

Two reaction rate constants of the release of MB from the aerogel matrix into the phosphate buffer solution were determined: *k*_1_ applies to the initial section (time interval from 0 to 5 min after placing the aerogel in the buffer) of the kinetic curve of the dye release into the buffer solution, when relaxation processes occur at the interphase boundary in the system; *k*_2_ applies to the section of the kinetic curve (time interval from 5 min to 30 min after placing the aerogel matrix in the buffer) when a steady-state flow of the MB release into the buffer solution is established. The recorded deviations from first-order kinetics in all cases considered in the work led to errors in the calculated values of the *k*_1_ and *k*_2_ rate constants that did not exceed 5%.

### 2.5 Activity in photogeneration of singlet oxygen

The activity of MB introduced into alginate aerogels (samples with linear dimensions of 2 cm × 1 cm and a thickness of 150 µm ± 50 μm) in the photogeneration of singlet oxygen ^1^O_2_ in air and in a phosphate buffer was determined by the intensity of ^1^O_2_ luminescence in the near-infrared region (*λ* = 1,267 nm). Luminescence was recorded using a Horiba Fluoromax Plus spectrofluorimeter (United States) with a DSS-IGA020L detector. To determine the MB activity during the ^1^O_2_ generation in air, aerogels with an MB content of 0.08–0.14 wt% and a PVP content in hybrid aerogels up to 25 wt% were excited by light with a wavelength of 650 nm. The ^1^O_2_ luminescence intensity (*λ* = 1,267 nm) was calculated as the average value for 10 measurements for each sample; the measurement error was 8%. To determine the activity of MB during the generation of ^1^O_2_ PBS in deuterated water, the samples were placed in a cuvette with a freshly prepared buffer of 3 mL. The solution was intensively stirred with a magnetic stirrer. When the concentration of MB in solution reached 5 × 10^-6^ M (monitored by the optical density of the MB absorption band with *λ* = 665 nm) (approximately 20 min), the aerogel film was removed. The resulting solution containing 5 × 10^−6^ M of MB was excited by light with *λ* = 665 nm, and the luminescence intensity of ^1^О_2_ was recorded at *λ* = 1,267 nm. The luminescence intensity of ^1^О_2_ was calculated as the average value for three measurements; the measurement error was 10%.

### 2.6 Surface structure of alginate aerogels

The surface structure of alginate aerogels was studied using atomic force microscopy (AFM) and scanning electron microscopy (SEM). The topography of aerogel surface areas was studied using an atomic force microscope (Russia, NT-MDT) with ETALON probes (Russia, NT-MDT). For each sample, 5–7 images measuring 10 × 10 μm^2^ were analyzed in the semi-contact mode.

The local Young’s modulus of the aerogels was measured using a BioScope Resolve atomic force microscope (Bruker, United States) in the force volume mapping mode. An RTESPA-150 probe with calibrated values of the stiffness constant *k*_r_ = 2.258 N/m and a probe radius of RT = 18.7 nm was used for shooting. Local stiffness distribution maps measuring 80 × 80 μm^2^ with a resolution of 40 × 40 points were obtained. Averaging was performed over 5–6 images for each sample. The resulting maps were processed using the NanoScope Analysis 1.8 software.

The structure of transverse cleavages of aerogel films was studied using a Melytec SV 32 scanning electron microscope (Russia), high vacuum mode, accelerating voltage 15–20 kV.

### 2.7 Differential thermal analysis (DTA)

The influence of the gelation method and the presence of PVP on the structure of the aerogels was determined by DTA on an NETTZCH STA 449 F3 synchronous thermal analyzer. The sample weights were 10–12 mg. The polymer destruction process was carried out with an air flow rate of 20 mL/min and a linear heating rate of 10 °C/min. The mass loss of the samples was recorded with an accuracy of 10^−3^ mg. The relative errors of temperature measurement and thermal effects were ±1.5 °C and ±3%, respectively. The destruction process was described by the dependences of mass loss (ML), mass loss rate (MLR), and thermal effects (DSC) on temperature. The onset temperature of mass loss (*T*_bml_), the onset temperature of the oxidation (*T*_oxb_), the total thermal effect of the process (*Q*, kJ/g), and the maximum rate of mass loss (*W*_max_) were determined.

### 2.8 Fourier-transform infrared spectroscopy (FTIR) analysis

FTIR analysis of the samples (aerogels of AA, CaA, and PVP, as well as hybrid polysaccharide-PVP aerogels) was performed using a Spectrum Two FT-IR Spectrometer (PerkinElmer, Waltham, MA, United States) in the attenuated total reflectance (ATR) mode. The spectrometer features were as follows: high-performance room temperature LiTaO_3_ MIR detector, standard optical system with KBr windows for data acquisition over a spectral range of 1900–1,200 cm^−1^ with a resolution of 0.5 cm^−1^. Spectra were normalized by the bands 1733 cm^−1^, 1,660 cm^−1^, 1,650 cm^−1^, and 1,599 cm^−1^.

### 2.9 Cell culture and cytotoxicity assessment

Primary culture of mesenchymal stromal cells (MSCs) isolated from human stromal vascular fraction (SVF) was used to evaluate the biocompatibility of aerogels *in vitro*. Cells were provided by the Sechenov University Biobank, which obtained tissue samples from healthy donors who had signed informed consent and ensured anonymization of the samples.

The cells were seeded into wells of a 96-well plate at a rate of 5,000 cells per well in a DMEM with L-glutamine (BioloT, art. 1.3.6) containing 10% fetal calf serum (FCS) (BioloT, Russia).

Samples of membranes that were sterilized using gamma irradiation were used to prepare extracts. These membrane samples were incubated for 24 h at 37 °C in an atmosphere of 5% CO_2_. Then, serial dilutions of the extract were prepared. Sodium dodecyl sulfate (SDS) detergent dilutions in the concentration range of 0.006–1.5 mg/mL were used as a positive control to artificially induce suppression of metabolic activity and cell death. A negative control was also provided—a series of wells into which a culture medium containing no extract of the test sample or SDS was added. The plates with extracts were then incubated for 24 h at 37 °C in an atmosphere of 5% CO_2_. After incubation, the reagent for determining the cell viability, alamarBlue (Thermo Fisher Scientific, United States), was added to the wells at a dilution of 1:10 in DMEM/F-12 without L-glutamine. The plate was incubated for 2 h at 37 °C in an atmosphere of 5% CO_2_. The fluorescence intensity of the dye was measured using a VICTOR Nivo fluorescent plate reader (PerkinElmer) at an excitation wavelength of 530 nm and an emission wavelength of 580 nm. The percentage of living cells was calculated as the ratio of the fluorescent signal recorded in the wells containing the test samples to that in the control wells.

A quantitative analysis of double-stranded DNA in the wells of the plate was performed as a reference test. For this purpose, cell lysis was performed by three repeated freeze–thaw cycles. The samples were frozen at −80 °C for 30 min/cycle. A working 1X TE buffer (composition: 10 mM Tris-HCl, 1 mM EDTA, pH 7.5), a stock DNA solution with a concentration of 2 mg/mL, 50 mg/mL, and a series of standard dilutions of λ-phage DNA for constructing a standard curve were prepared. A 100-μL aliquot of the obtained cell lysate or a standard dilution of λ-phage DNA, as well as 100 μL of PicoGreen dye (Thermo Fisher Scientific, P7589), was used to construct a calibration curve. The plate was incubated for 15 min at room temperature in the dark, and then the fluorescent signal in the wells was measured using the following signal acquisition parameters: excitation wavelength 480 nm, emission wavelength 530 nm. The obtained data were used to construct a calibration curve and then calculate the DNA concentration in the wells with the resulting cell lysate.

Primary cultures of mesenchymal stromal cells (MSCs) isolated from the human stromal vascular fraction (SVF) were used to evaluate the biocompatibility of aerogels *in vitro*. The cells were seeded into the wells of a 96-well plate at a density of 5,000 cells per well in DMEM cell culture medium with L-glutamine (BioloT, art. 1.3.6), supplemented with 10% fetal bovine serum (FBS) (BioloT, Russia).

Aerogels, sterilized using gamma irradiation, were used to prepare extracts. These samples were incubated for 24 h at 37 °C in an atmosphere containing 5% CO_2_. Subsequently, serial dilutions of the extract were prepared. Sodium dodecyl sulfate (SDS) detergent dilutions in the concentration range of 0.006 mg/mL to 1.5 mg/mL were used as a positive control to artificially induce suppression of metabolic activity and cell death. A negative control was also included, consisting of a series of wells containing culture medium without the test sample extract or SDS.

The plates with extracts were then incubated for an additional 24 h at 37 °C in an atmosphere of 5% CO_2_. After incubation, alamarBlue reagent (Thermo Fisher Scientific, United States) was added to the wells at a dilution of 1:10 in DMEM/F-12 without L-glutamine. The plate was incubated for 2 h at 37 °C in an atmosphere of 5% CO_2_. Following this, the fluorescence intensity of the dye was measured using a VICTOR Nivo fluorescent plate reader (PerkinElmer) at an excitation wavelength of 530 nm and an emission wavelength of 580 nm. The percentage of living cells was then calculated as the ratio of the fluorescent signal recorded in the wells containing the test samples to that in the control wells.

A quantitative analysis of double-stranded DNA in the wells of the plate was performed as a reference test. For this purpose, cell lysis was achieved through three repeated freeze–thaw cycles, with freezing conducted at −80 °C for 30 min per cycle. A working 1X TE buffer (composed of 10 mM Tris-HCl, 1 mM EDTA, pH 7.5), a stock DNA solution with concentrations of 2 mg/mL and 50 mg/mL, and a series of standard dilutions of λ-phage DNA for constructing a standard curve were prepared. Then, 100 μL of the obtained cell lysate or a standard dilution of λ-phage DNA was combined with 100 μL of PicoGreen dye (Thermo Fisher Scientific, P7589) to construct the calibration curve. The plate was incubated for 15 min at room temperature in the dark, after which the fluorescent signal in the wells was measured using the following acquisition parameters: excitation wavelength of 480 nm and emission wavelength of 530 nm. The obtained data were used to construct a calibration curve and subsequently calculate the DNA concentration in the wells containing the resulting cell lysate.

## 3 Results and discussion

### 3.1 Surface structure of alginate and hybrid aerogels (based on AFM and SEM data)

**FIGURE 1:**
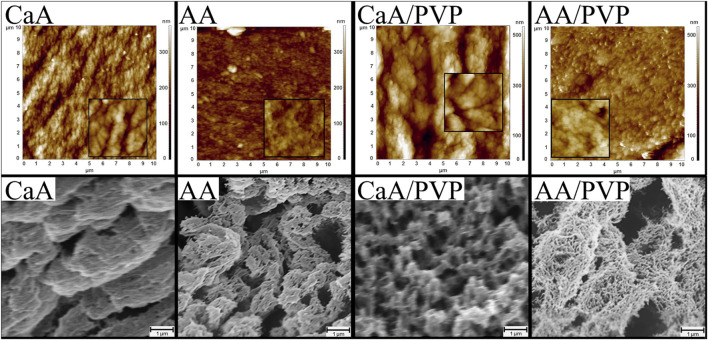
AFM images (top row) of the surface areas. Image size is 10 × 10 μm^2^, and inset size is 1 × 1 μm^2^. SEM images (bottom row) of the cleavage surface of alginate aerogels (CaA, AA) and hybrid aerogels (CaA/PVP and AA/PVP).

After drying in a sc-CO_2_, the alginic acid (AA) and calcium alginate (CaA) aerogels were opaque, uncolored films with a thickness of 100–1,000 μm. The structure of the CaA aerogels appears to be more organized and densely packed than the structure of alginic acid aerogels (Figure 1).

As follows from AFM and SEM images, the structure of the aerogels cross-linked with calcium chloride (Figure 1, CaA) is characterized by a dense packing of lamella-like formations, with a lamella thickness of approximately 80 nm (as seen in the inset showing an enlarged fragment). In contrast, the surface of the alginic acid aerogel (Figure 1, AA) consists of fine-grained particles without any pronounced overall structure.

The introduction of PVP into the aerogel, which phase separates and is presumably localized on the surface of the alginate layers, results in visible thickening of the alginate lamellae (according to AFM data) and a loosening of the polysaccharide structure (according to SEM data). This led to disordering of the structure and, as will be shown below, an increase in the system’s porosity. The SEM images of the cleavage surface of the aerogels shown in Figure 1 (bottom row) confirm the increased porosity of hybrid aerogels on the basis of alginate and PVP, compared to monocomponent aerogels. At the same image size, the porous structure of AA aerogels (Figure 1, AA) and the superporous structure of the AA/PVP aerogels (Figure 1, AA/PVP) is visible, which determines the above-mentioned density of aerogels and their high vapor and gas permeability.

### 3.2 IR spectra of alginate and hybrid aerogels

**FIGURE 2:**
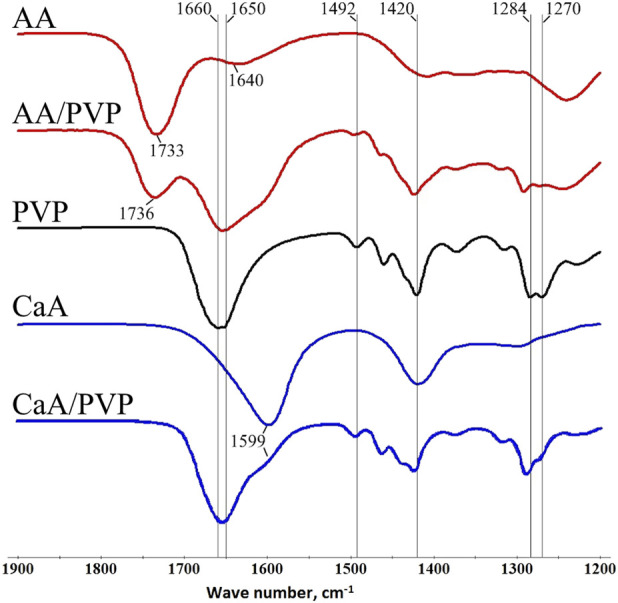
IR spectra of the AA aerogel, hybrid AA/PVP aerogel (16 wt% PVP), PVP, CaA aerogel, and hybrid CaA/PVP aerogel (14 wt% PVP).

IR spectroscopy was used to describe the features of the chemical structure of aerogel and the possible formation of PVP–aerogel complexes. Figure 2 shows the IR spectra (transmission) of alginate aerogels and hybrid alginate-PVP aerogels. Supplementary Table S1 provides data on the correspondence of bands to certain groups in polymers.

The spectrum of alginic acid aerogel contains a peak at 1733 cm^−1^ (curve AA), corresponding to the stretching vibrations of carbonyl groups C=O in aliphatic acids; in the AA/PVP aerogel (curve AA/PVP), this band shifts to 1736 cm^−1^, possibly due to the formation of hydrogen bonds with fragments of PVP macromolecules (see Figure 5). A low-intensity peak corresponding to vibrations of carboxylate ions (1,640 cm^−1^) is also observed in the spectrum of the AA aerogel, which in the presence of PVP overlaps with the peak at 1,650–1,660 cm^−1^, corresponding to vibrations of the C=O groups of the pyrrolidone ring. In this case, as can be seen from Figure 2 (curves AA, AA/PVP), the maximum of the integral peak is in the region of 1,650–1,660 cm^−1^. It should also be noted that the peaks in the region of 1,420–1,492 cm^−1^, which correspond to the deformation vibrations of the CH, CN, NH groups of PVP macromolecules (curve PVP), also shift by 3–4 cm^−1^. However, the greatest changes are observed for the peaks at 1,270 cm^−1^ and 1,284 cm^−1^, corresponding to the vibrations of the CH and CN groups: not only do their maxima shift by 8–9 cm^−1^, but the ratio of their intensities also changes (Figure 2, curve AA/PVP).

For the CaA/PVP hybrid aerogel (curve CaA/PVP), the maximum of the integral peak corresponding to the stretching vibrations of the COO- groups of the polysaccharide (in CaA, the peak is observed at 1,599 cm^−1^, curve CaA) and the C=O groups of PVP is also in the region of 1,650–1,660 cm^−1^ (Supplementary Table S1). At the same time, the maximum of the peak at 1,418 cm^−1^, corresponding to vibrations of the carboxylate groups of the polysaccharide, shifts in the presence of PVP by 6 cm^−1^. In addition, the peaks at 1,270 cm^−1^ and 1,284 cm^−1^, corresponding to vibrations of the CH and CN groups, also shift by 5–6 cm^−1^.

The above changes indicate the formation of intermolecular hydrogen and hydrophobic bonds in the polysaccharide (AA, CaA)-PVP systems, which are responsible for the structure of the corresponding hybrid aerogels. In this case, in PVP macromolecules, the CN and NH groups take an active part in this process by forming hydrogen bonds with the C=O and COO- groups of the polysaccharide.

It should also be noted that the presence of a band (1,640 cm^−1^) corresponding to vibrations of carboxylate ions in the IR spectrum of AA aerogels is obviously associated with the presence of a residual amount of sodium alginate units in the AA macromolecules. Indeed, according to elemental analysis data, a certain amount of sodium ions remains in the samples of AA and CaA aerogels (Table 1); that is, a small part of the links (apparently, residues of mannuronic acid) of the macromolecules of alginic acid and sodium alginate remain uncrosslinked (Bajas et al., 2021; Volkova et al., 2019).

**TABLE 1:** Sodium content in alginic acid and calcium alginate aerogels, as well as in the original sodium alginate (according to elemental analysis).

Sample	Na, wt%	Degree of substitution of Na, %
Original SA	8.6	0
AA aerogel	0.7	91.9
CaA aerogel	0.2	97.7

### 3.3 Effect of PVP on porosity and swelling of alginate aerogels

As already mentioned, aerogels are mesoporous materials with a large specific surface area.

The mesoporous structures of the AA and CaA aerogels were formed during the drying process of the corresponding alcogels in sc-CO_2_, which in turn were obtained by treating alginate hydrogels with isopropyl alcohol.

As described above, alginate hydrogels were produced from aqueous solutions of sodium alginate (SA) in two ways: by treatment with hydrochloric acid or with calcium chloride. In both cases, water-insoluble alginic acid (AA) and calcium alginate (CaA), respectively, were formed. Both polymers existed in water as hydrogels.

**FIGURE 3:**
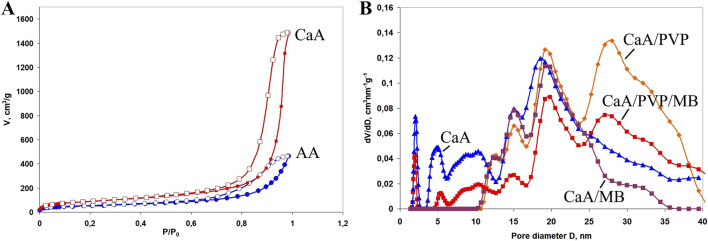
**(A)** Isotherms of nitrogen adsorption (filled symbols) and desorption (empty symbols) on the surface of AА and CaА aerogels. **(B)** Pore size distribution for calcium alginate aerogels in the nanometer range, where the curve is calcium alginate, CaA/MB, CaA/PVP, and CaA/PVP/MB. The content of PVP in the samples is 2.5 wt%, and the content of MB is 0.1 wt%.

The surface area and pore sizes of the samples confirm the mesoporous structure of the aerogels. Figure 3A shows the nitrogen adsorption and desorption isotherms for the AA and CaA aerogels. The isotherms were obtained by the low-temperature nitrogen sorption method (77.4 K) and form a hysteresis in the range of relative pressures of 0.70–0.99, indicating capillary condensation of nitrogen in the mesopores. This type of isotherm is typical for mesoporous samples and can be classified as type IV according to the IUPAC classification. The sorption isotherms of other studied aerogels demonstrate similar behavior, which indicates a developed porous structure of all studied samples.

For detailing the porous structure of all the studied aerogels and to acquire data on their specific surface area and porous structure parameters in the entire range of micro- and mesopores, the quenched solid density functional theory (QSDFT) method based on the density functional theory was used (Gor et al., 2012; Neimark et al., 2009). QSDFT models for N_2_ adsorption account for a variety of pore geometries, including slit micropores (typical of activated carbon materials), cylindrical pores (typical of micro- and mesopore channel models), and spherical pores (typical of some carbon materials prepared by template synthesis). A library of QSDFT models is available in the software for the equipment used. In addition to the positron annihilation method, we also used the t-plot–De Boer method to estimate the contribution of micropores in the presence of mesopores. Table 2 presents data on the specific surface area and porous structure parameters of aerogels under the assumption of a cylindrical model of pore geometry. Figure 3B shows the pore size distribution for some of the studied aerogels. As can be seen from Table 2, aerogels cross-linked with Ca^2+^ have a higher specific surface area and a larger pore volume than alginic acid aerogels (samples 1 and 2 in Table 2). This may be due to the greater affinity of carbon dioxide molecules for calcium ions, which are the cross-linking centers of calcium alginate macromolecules, which facilitates pore formation in CaA aerogels in an sc-CO_2_ environment (Nikitin et al., 2006).

**TABLE 2:** Specific surface area and parameters of the porous structure of aerogel systems based on sodium alginate.

№	Sample	*S*_BET_^а^, m^2^/g	*V*_t_^b^, cm^3^/g	*V*_DFT_^c^, cm^3^/g	*S*_DFT_^c^, m^2^/g	*V*_mic_^d^, cm^3^/g	*S*_mic_^d^, m^2^/g
1	AA	248	0.80	0.59	227	0.005	16
2	CaA	384	2.41	2.05	381	0.007	22
3	AA/PVP*	308	1.06	0.82	271	0.010	30
4	CaA/PVP	441	2.91	2.47	383	0.015	44
5	CaA/MB	244	1.42	1.18	240	0.016	39
6	AA/MB	174	0.40	0.32	161	0.011	26
7	CaA/PVP/MB	325	2.19	1.51	301	0.010	28
8	AA/PVP/MB	185	0.54	0.42	168	0.010	24

*–The PVP content in aerogels is ∼16 wt%; the MB content is 0.1 wt%; а–specific surface area of aerogels (BET); b–total pore volume at *P/P_0_* = 0.99; c–pore volume and area (DFT); d–volume and area of micropores (t-plot).

It should also be noted that the introduction of PVP into the composition of aerogels based on AA and CaA increases both the *S*_BET_ and the total pore volume (*V*_t_) of both types of aerogels by 30%–50%.

In reality, in these systems, PVP exhibits the properties of a pore-forming agent. As shown by Elessawy et al. (2021) and Kononova et al. (2016), this may be due to the fact that PVP can be removed from polymer compositions during washing of the hydrogel with water or during the preparation of the alcogel (see the Materials and Methods section). At the same time, according to elemental analysis Section 2.2.2, not all PVP is washed out from the hydrogels. A certain amount of PVP is still present in hybrid aerogels (Table 2). It is believed that intermolecular complexes are formed due to the acid–base interaction between the amine groups of PVP and the carboxyl groups of SA. Their possible structure is shown in Supplementary Figure S1A (Elessawy et al., 2021). In addition, the formation of hydrogen bonds is possible during the interaction of the carbonyl groups of PVP with the hydroxyl groups of sodium alginate (Supplementary Figure S1B) (Çaykara et al., 2007).

**FIGURE 4:**
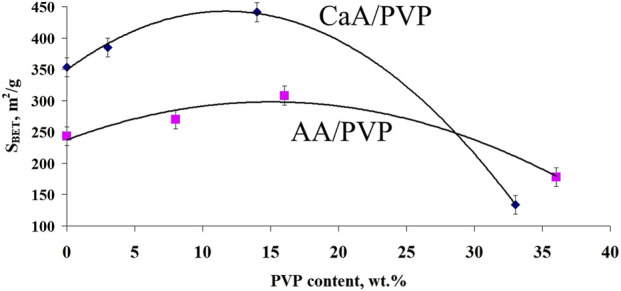
Dependence of the specific surface area of hybrid aerogels on the PVP content for AA/PVP and CaA/PVP.

This is also confirmed by the data on the distribution of pore sizes in the studied aerogels (Figure 3B). In particular, it is shown that new mesopores with sizes of approximately 26–28 nm are formed in the CaA/PVP aerogel, which were absent both in the original CaA aerogel and in the aerogel with introduced MB (curves CaA, CaA/MB, and CaA/PVP). Such pores are also preserved in three-component aerogels (CaA/MB/PVP) (Figure 3B, curve CaA/PVP/MB). Therefore, despite its partial leaching from the composition, the idea of PVP as a pore-forming agent in CaA and AA hydrogels due to the formation of weakly bound complexes with sodium alginate turns out to be quite justified. Such weakly bound complexes are probably responsible for the formation of the structure of gels and aerogels of calcium alginate (or alginic acid) and PVP. The extreme nature of the observed dependence of *S*_BET_ for CaA/PVP and AA/PVP aerogels on the content of the amphiphilic polymer also becomes clear (Figure 4). Moreover, in both cases shown in Figure 4, the maximum specific surface is observed for aerogel with a PVP content of ∼15%.

The use of the positron annihilation method made it possible to establish that PVP promotes the formation of micropores (with a characteristic size of fractions of nm) in aerogels based on alginic acid and calcium alginate. This follows from Table 3, which presents data on the sizes of micropores for AA aerogels with and without PVP; the data were obtained by studying the distribution of positron annihilation radiation over time (Consolati et al., 2023; Shantarovich et al., 2002; Shantarovich et al., 2019). As shown in Table 3, two groups of low-intensity micropores (*I*_3_, *I*_4_) are formed in the system with average values of *R*_3_ and *R*_4_ pore radii, which correspond to the *τ*_3_ and *τ*_4_ lifetimes of localized positronium relative to annihilation. It should be noted that, in accordance with the measurements carried out, the *R*_3_ and *R*_4_ pore radii change noticeably, from 0.15 nm to 0.33 nm for the original sample and up to 0.18 nm and 0.6 nm for the sample subjected to the action of the “loosening agent” (PVP).

**TABLE 3:** Effect of PVP on the micropore sizes of the AA aerogel determined by the positron annihilation method.

Sample	*S*_BET_, m^2^/g	*τ*_3_^a^, ns	*I*_3_^b^, %	*τ*_4_^a^, ns	*I*_4_^b^, %	*R*_3_^c^, nm	*R*_4_^c^, nm
AA	248	0.83 ± 0.11	7.36 ± 1.10	2.68 ± 0.16	3.83 ± 0.46	0.15	0.33
AA/PVP*	308	1.13 ± 0.08	6.00 ± 0.48	7.37 ± 0.68	1.88 ± 0.13	0.18	0.60

*–The PVP content in the aerogel is ∼16 wt%; a–lifetimes of localized positronium relative to annihilation; b–intensities of the micropore signal; c–average values of the pore radii.

Thus, the microporosity (as well as the mesoporosity) of the studied materials increases with the introduction of PVP. The increase in the porosity of CaA aerogels compared to alginic acid aerogels (Table 2) is also evidenced by the values of the local Young’s modulus for these systems (690 MPa and 430 MPa, for AA and CaA, respectively), reflecting the local rigidity of the material. Moreover, the introduction of PVP into aerogels, which acts as a pore-forming agent in these systems, significantly reduces the rigidity of both aerogels (∼ to 40 MPa and 25 MPa, respectively).

The order of concentration of micropores according to the annihilation data in the studied samples can be estimated under the assumption that for a positronium atom to enter a pore, the distance between the pores should not exceed the diffusion path length (*l*_D_) of the positronium atom before localization in the pore.

$$l_{D}={\left(D_{\text{Ps}}\times \tau_{f}\right)}^{1/2}$$

With a known diffusion coefficient *D*_Ps_ ∼ 10^−4^ cm^2^/s and a lifetime of non-localized positronium relative to annihilation *τ*_f_ ∼ 0.3 ns, an estimate for the concentration of micropores can be found:

$$C_{\text{mic}}\approx l_{D}^{-3}={\left(D_{\text{Ps}}\times \tau_{f}\right)}^{-3/2}\approx 2\times 10^{20}\;{\text{cm}}^{-3}$$

Taking into account the average radius of micropores of the order of ∼0.5 nm (Table 3), the volume of one micropore *v* = 0.5 × 10^−21^ cm^3^. In this case, the total volume of micropores, *V*_mic_, is calculated by the following equation:

$$V_{\text{mic}}\left[\frac{{\text{cm}}^{3}}{g}\right]=v\times C_{\text{mic}}\times \rho^{-1}$$

with the values of wall densities *ρ* ≈ 1 g/cm^3^ close to the values of *V* ≈ 10^−2^ cm^3^/g presented in Table 2, which were retrieved by the sorption method.

Thus, a certain correlation between the results of sorption and annihilation methods is observed.

The ability of aerogels to swell in PBS buffer was studied. Supplementary Table S2 presents the *Q* growth data over an hour. As can be seen from the table, the *Q* of ACa aerogels (∼1,480%) is higher than that of alginic acid aerogels (∼1,100%). In the presence of PVP, the swelling ratio of ACa and AA aerogels increases slightly (*Q* is 1700% and 1,500% respectively). This may be due to the loosening effect of PVP on the structure of alginate, which is confirmed by AFM and SEM data.

### 3.4 Thermogravimetry

**FIGURE 5:**
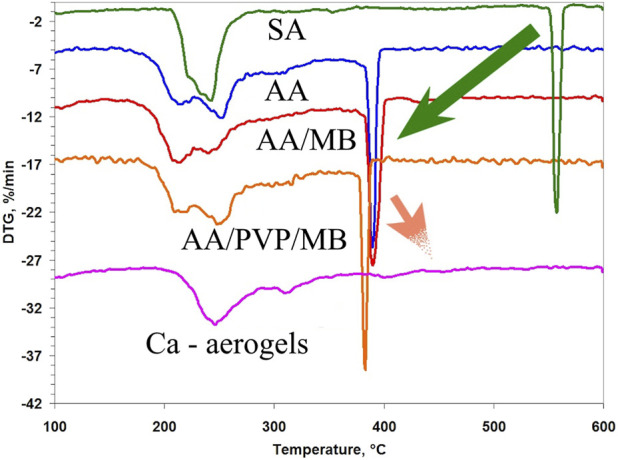
DTG curves of alginic acid and calcium alginate aerogels and their complexes with PVP: initial SA; AA, AA/MB, AA/PVP/MB; and general view of curves for CaA, CaA/MB, and CaA/PVP/MB.

Figure 5 shows the differential thermogravimetric (DTG) curves of aerogels based on calcium alginate and alginic acid.

The first thing that is revealed in these data is the lower thermal stability of the aerogels based on AA compared to the CaA aerogels. As follows from Table 4, the onset temperature of destruction (*T*_onset_) of all AA aerogels is lower than the corresponding *T*_onset_ for CaA aerogels by approximately 20 °C.

**TABLE 4:** DTA parameters of CaA and AA aerogels, as well as the initial SA polysaccharide.

№	Sample	*Т*_onset_ °C
1	SA	210
2	AA	185
3	AA/MB	183
4	AA/PVP/MB	195
5	AA/PVP	190
6	СаA	204
7	СаA/PVP	217
8	CaA/MB	209
9	CaA/PVP/MB	213

Moreover, the higher onset temperature of destruction of the hybrid CaA/PVP and CaA/PVP/MB aerogels (№ 7 and №. 9 in Table 4), compared to CaA aerogels in the absence of PVP (№ 6 in Table 4), as well as AA/PVP and AA/PVP/MB (№ 5 and №. 4 in Table 4), compared to AA aerogels (№ 2 in Table 4) may indicate the formation of hydrogen bonds between the carbonyl groups of PVP and the hydroxyl groups of sodium alginate (Solovieva et al., 2024) in PVP-SA mixtures.

It should be noted that the introduction of MB into AA and CaA aerogels does not affect the thermal destruction mechanism of alginate and hybrid aerogels.

### 3.5 Features of diffusion of MB immobilized in aerogels into PBS

**FIGURE 6:**
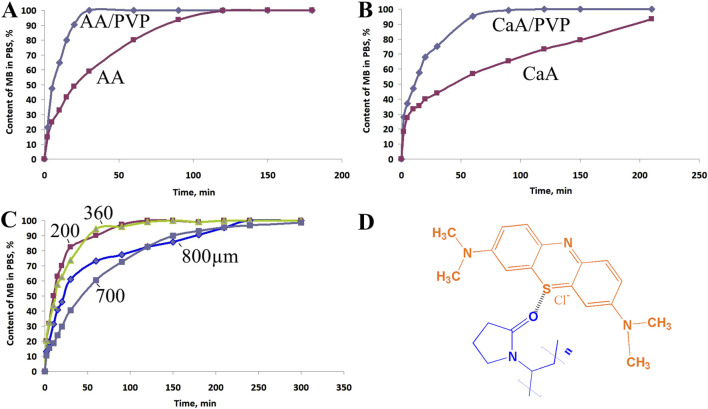
Kinetic curves of MB diffusion into PBS from **(A)** AA aerogels and **(B)** CaA aerogels produced in the absence and in the presence of PVP. The MB content in the samples is 0.1 wt%, and the PVP content is ∼2.5 wt%. **(C)** Dependences of the diffusion rate of MB from CaA aerogel samples of different thicknesses into PBS: 800 μm, 700 μm, 360 μm, and 200 μm. **(D)** Possible structure of MB-PVP complexes.

Figure 6 shows the dependence of the concentration of MB released from AA-based aerogel films (Figure 6A) and CaA aerogel (Figure 6B) in a buffer solution (PBS) in the absence of PVP and in its presence. Figure 6C shows the dependence of the diffusion rate of MB from CaA aerogels of different thickness (200–800 μm) into PBS. Figure 6D shows the possible structure of MB - PVP complexes.

The rate constants and release times of 50% and 100% of methylene blue for AA and CaA aerogels are given in Table 5. It is evident that the process of MB diffusion from the AA and CaA aerogels into the phosphate buffer occurs in at least two stages with a decreasing rate. At the same time, for both types of gels, the influence of PVP on the kinetics of the MB release, especially at the second stage of the process, is observed.

**TABLE 5:** Parameters of the dye diffusion from aerogels into phosphate buffer (PBS). The MB content in the samples is ∼0.5 wt%.

Sample	PVP content in aerogels, wt%	*k*_1_ × 10^2^,s^−1^	*k*_2_ × 10^3^,s^−1^	Time to 50% MB diffusion, min
AA/MB	-	7.2	1.2	20
AA/MB/PVP	2.5%	7.3	2.2	15
CaA/MB	-	7.0	0.6	40
СаA/MB/PVP	2.5%	7.4	0.8	10

It can be seen that the rate constant *k*_2_ of MB diffusion from AA aerogels is higher than the corresponding constant of diffusion from CaA aerogels, possibly due to the partial solubility of acidic aerogels in a phosphate buffer. In addition, the constants of the MB diffusion rate from hybrid aerogels (based on alginate and PVP) exceed (Table 5) the values of the rate constants for the corresponding single-component polysaccharide gel. This is obviously due to the increased porosity of the hybrid aerogels. In addition, MB in hybrid aerogels can be localized near PVP macromolecules, forming weakly bound complexes with the polymer. In this case, the dye will be released into the buffer at a higher rate due to the good solubility of PVP in aqueous media. The formation of weakly bound MB–PVP complexes in aqueous solutions was previously observed using H^1^NMR (Kuryanova et al., 2023).

It should also be noted that the thickness of the sample has little effect on the diffusion rate of the dye: with a fourfold increase in the thickness of the sample, the diffusion rate slightly decreases (Figure 6C; Supplementary Table S3), which may indicate kinetic difficulties at the interphase boundary when the dye is released into the solution.

In addition, it is shown that the dye concentration has virtually no effect on the MB diffusion rate at the first stage of the process. At the second stage of the process, when the MB concentration in the buffer increases, the dye diffusion rate slows down somewhat due to concentration difficulties. In other words, the main factor determining the rate of dye diffusion is the structure of the aerogel. Previously, we observed a similar effect of the structure of polymer carriers on the rate of methylene blue diffusion into buffer solutions when studying the effect of the nature of the alginate solid gel matrix on dye diffusion (Solovieva et al., 2024). At the same time, PVP decreased the rate of dye release from the alginate xerogel into the phosphate buffer. This was probably due to the formation of strong cross-linked polysaccharide-PVP-MB complexes, which hinder the release of immobilized MB into the buffer. Thus, by changing the structure of hybrid aerogels, it is possible to control the kinetics of dye diffusion.

### 3.6 Activity of MB immobilized in aerogels in the photogeneration of singlet ^1^О_2_ oxygen

**FIGURE 7:**
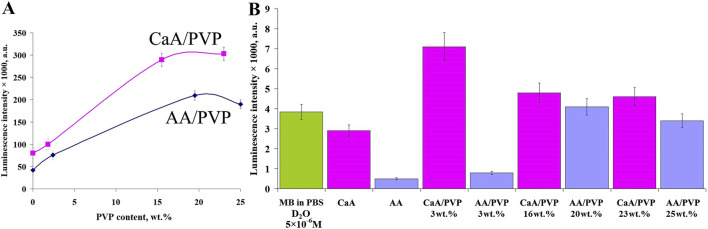
**(A)** Dependence of the luminescence intensity of ^1^О_2_ (*λ* = 1,267 nm) in air upon photoexcitation of aerogels containing MB (AA/PVP, CaA/PVP) on the PVP content. **(B)** The luminescence intensity of ^1^O_2_ in a phosphate buffer (D_2_O) after diffusion of MB from aerogels with different PVP content. The MB content in the samples is 0.1 wt%.

As noted above, the activity of the dye immobilized in aerogels in generating singlet oxygen was studied by the luminescence of ^1^О_2_ upon photoexcitation of MB in air and after diffusion of the dye into a buffer solution (Figures 7A,B).

It is evident that the activity of MB (introduced into aerogels) in the photogeneration of ^1^О_2_ depends on the nature of the aerogel, its structure, and its porosity, regulated by the presence of PVP. In particular (Figure 7A), the activity of MB introduced into CaA aerogels is greater than that in AA aerogels. As can be seen from Figure 7A, the sequence of activity of MB introduced into aerogels of different structures in the generation of singlet oxygen in air (upon photoexcitation of aerogels containing MB) is as follows: CaA/PVP > AA/PVP > CaA > AA, that is, the lowest activity of MB is observed when MB is introduced into a single-component aerogel. The use of a matrix of hybrid aerogels containing PVP (AA/PVP) increases the MB activity up to four times.

The same is observed in CaA aerogels. In the hybrid CaA/PVP aerogel, the MB activity increases more than threefold compared to MB immobilized in CaA aerogels. This pattern is valid for the excitation of MB in the composition of solid aerogels (Figure 7A) and for MB released into a phosphate buffer (Figure 7B). Obviously, this is due to the localization of MB when it is introduced into hybrid aerogels mainly in the PVP phase, which, as we have previously shown (Kuryanova et al., 2023), forms weakly bound complexes with MB. This promotes disaggregation of the dye and an increase in the activity of MB in the photogeneration of singlet ^1^О_2_ oxygen. Obviously, the dye diffuses into the phosphate buffer in a complex with PVP. At the same time, as follows from Figure 7B, there is an optimal concentration of PVP (15–17 wt%), above which the effect of the amphiphilic polymer on the rate of the photogeneration process decreases in the same way as the effect of the PVP content in the aerogels on their porosity (Figure 4).

### 3.7 Cytotoxicity assessment

**FIGURE 8:**
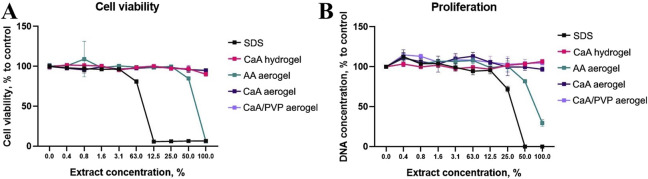
Cell viability **(A)** and proliferation activity **(B)** assessment for CaA hydrogel and AA, CaA, and CaA/PVP aerogels; SDS is used as the control.

As illustrated in Figure 8, the evaluation of the biocompatibility of various hydrogel (CaA) and aerogel (AA, CaA, and CaA/PVP) samples revealed that almost all samples were non-harmful to MSCs, except for the AA aerogel. For this particular sample, the most concentrated extract demonstrated a significant reduction in metabolic activity, comparable to that observed with the toxic sodium dodecyl sulfate (SDS) control.

Similar findings were obtained from the proliferation assessment, which indicated that the CaA and CaA/PVP sample aerogels did not exhibit any toxic effects and did not adversely affect cell physiology. In contrast, the AA aerogel resulted in a marked suppression of both metabolism and proliferation. These effects observed may be attributed to the influence of alginic acid, which could induce a decrease in pH, thereby negatively impacting cell health and functionality. It was confirmed via the evaluation of the culture medium colour (Supplementary Figure S2). These results suggest that while most aerogels are biocompatible, careful consideration must be given to the formulation of AA aerogels due to their potential cytotoxic effects.

## 4 Conclusion

We developed and characterized aerogels based on AA and CaA, including hybrid alginate/PVP aerogels, using AFM, SEM, thermogravimetry, and IR spectroscopy. The specific surface area and pore size distribution of the aerogels were determined by low-temperature nitrogen sorption. We employed the positron annihilation lifetime spectroscopy method to detail the porous structure of all studied aerogels. This approach revealed that PVP acts as a pore-forming agent, increasing the specific surface area by 30%–50% compared to single-component polysaccharide aerogels and creating new meso- and micropores on the nanometer scale. This enhanced porosity determines the increased permeability of the matrices to air and water vapor, which can promote wound ventilation.

The primary factor controlling the MB release rate from the alginate aerogels is their structure and chemical composition. The dye diffusion from AA aerogels was higher than from CaA aerogels. Compared to single-component alginate aerogels, the hybrid PVP-containing aerogels demonstrated a 1.5 to 2 times faster dye release into the buffer solution. This acceleration is attributed to the increased porosity and the probable formation of water-soluble PVP-MB complexes, facilitating methylene blue transfer into the aqueous medium.

The activity of MB embedded in the aerogels in the photogeneration of singlet oxygen (^1^O_2_) depended on the aerogel’s nature, structure, and porosity, which are regulated by the presence of PVP. The sequence of MB photoactivity in singlet oxygen generation in air was as follows: CaA/PVP > AA/PVP > CaA > AA. Using hybrid PVP-containing aerogels as a matrix increased the MB photoactivity by up to four times. A similar enhancing effect of PVP was observed for methylene blue released from the aerogels into the buffer. This enhancement is likely due to PVP preventing MB aggregation by forming MB/PVP complexes, thereby improving the photosensitizer’s efficiency.

CaA and CaA/PVP aerogels were found to be non-cytotoxic. In contrast, AA aerogels exhibited some cytotoxicity, which we attribute to the influence of alginic acid potentially inducing a local decrease in pH, thereby negatively impacting cell health and functionality.

Thus, alginate/PVP hybrid aerogels are promising matrices for creating medicinal forms of photosensitizers like methylene blue for the photodynamic therapy of difficult-to-heal purulent wounds and trophic ulcers.

## Data Availability

The original contributions presented in the study are included in the article/Supplementary Material, further inquiries can be directed to the corresponding authors.
