# Supplementary material for: Photosensitizing systems based on alginate aerogels and methylene blue for controlled release of dye for antimicrobial photodynamic therapy
Source: Front Chem. 2025 Nov 11;13:1702876. doi: 10.3389/fchem.2025.1702876 (PMC12643984; doi:10.3389/fchem.2025.1702876)
Supplement: Supplementary file 1 [file DataSheet1.pdf]

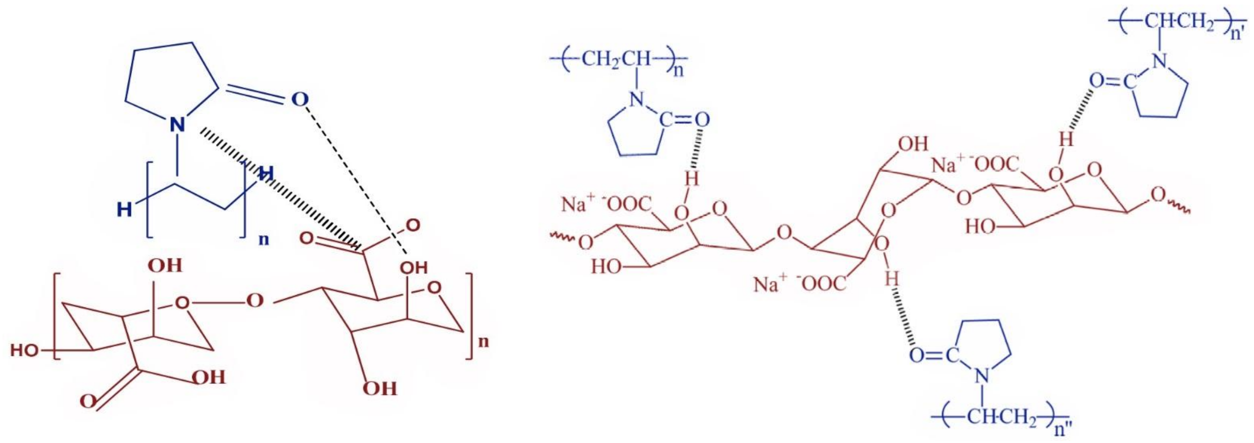

(A) (B)

Figure S1. Possible structures of the SA-PVP complex: (A) according to (Elessawy et al., 2021), (B) according to (Çaykara et al., 2007).

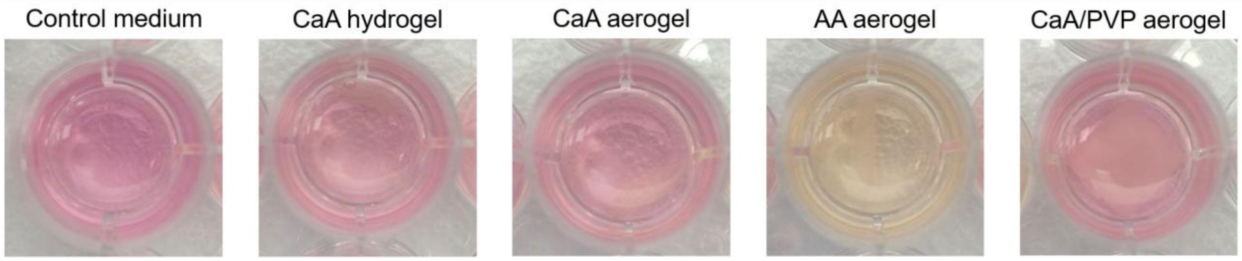

Figure S2. Culture medium color change after 24h incubation with alginate gels.

Table S1. Correlation of the main bands in the IR spectra of aerogels based on alginate and PVP.

Bands, cm^{-1}	Attribution
1733, 1736	*v* CO (COOH in AA)
1640, 1599, 1418-1424	*v* CO (COO- in AA and CaA)
1650	CO (C=O in PVP)
1420-1495	*σ* CH (aerogel and PVP), CN, NH (PVP)
1270-1289	*σ* CH (aerogel and PVP), CN (PVP)

Table S2. Kinetics of change in the swelling ratio (Q, %) for aerogel samples in a PBS at t=22 °C.
*PVP content ~16 wt.%

Time, min	Q, %			
	AA	AA/PVP*	CaA	CaA/PVP*
5	179.4	235.2	214.0	232.7
10	243.6	382.1	333.2	367.7
15	364.7	492.9	473.8	521.4
20	420.5	615.8	622.4	592.4
25	496.9	744.4	745.5	733.0
30	561.8	872.3	895.0	871.7
35	699.6	1020.1	1018.9	1001.8
40	833.7	1112.3	1098.3	1159.4
45	909.8	1236.2	1228.1	1289.1
50	973.1	1359.9	1308.1	1436.9
55	1077.6	1453.6	1385.7	1588.5
60	1172.6	1555.1	1478.9	1730.9

Table S3. Rate constants of MB diffusion from samples of CaA aerogels of different thicknesses into PBS.

Thickness, µm	k_1×10^2, s^{-1}	k_2×10^3, s^{-1}	Time to 50% MB diffusion, min	Time to 100% MB diffusion, min	[MB]×10^6, mol/g
200	7.4	1.2	12	90	3.2
360	6.9	1.4	10	90	0.9
700	7.2	0.9	45	210	2.4
800	6.8	1.5	25	210	1.8
